# Toward a fully implantable ecosystem for adaptive neuromodulation in humans: Preliminary experience with the CorTec BrainInterchange device in a canine model

**DOI:** 10.3389/fnins.2022.932782

**Published:** 2022-12-19

**Authors:** Gerwin Schalk, Samuel Worrell, Filip Mivalt, Alexander Belsten, Inyong Kim, Jonathan M. Morris, Dora Hermes, Bryan T. Klassen, Nathan P. Staff, Steven Messina, Timothy Kaufmann, Jörn Rickert, Peter Brunner, Gregory A. Worrell, Kai J. Miller

**Affiliations:** ^1^Department of Neurosurgery, Mayo Clinic, Rochester, MN, United States; ^2^Chen Frontier Lab for Applied Neurotechnology, Tianqiao and Chrissy Chen Institute, Shanghai, China; ^3^Department of Neurology, Mayo Clinic, Rochester, MN, United States; ^4^Department of Biomedical Engineering, Brno University of Technology, Brno, Czechia; ^5^Department of Neurosurgery, Washington University in St. Louis, St. Louis, MO, United States; ^6^National Center for Adaptive Neurotechnologies, Albany, NY, United States; ^7^Department of Neuroradiology, Mayo Clinic, Rochester, MN, United States; ^8^Department of Physiology and Biomedical Engineering, Mayo Clinic, Rochester, MN, United States; ^9^CorTec GmbH, Freiburg, Germany

**Keywords:** neuromodulation, brain–computer interface, neurotechnology, invasive, adaptive

## Abstract

This article describes initial work toward an ecosystem for adaptive neuromodulation in humans by documenting the experience of implanting CorTec's BrainInterchange (BIC) device in a beagle canine and using the BCI2000 environment to interact with the BIC device. It begins with laying out the substantial opportunity presented by a useful, easy-to-use, and widely available hardware/software ecosystem in the current landscape of the field of adaptive neuromodulation, and then describes experience with implantation, software integration, and post-surgical validation of recording of brain signals and implant parameters. Initial experience suggests that the hardware capabilities of the BIC device are fully supported by BCI2000, and that the BIC/BCI2000 device can record and process brain signals during free behavior. With further development and validation, the BIC/BCI2000 ecosystem could become an important tool for research into new adaptive neuromodulation protocols in humans.

## 1. Introduction

### 1.1. Adaptive neuromodulation

Neural interface technology allows us to measure activity from the nervous system and stimulate it, creating closed-loop systems that establish artificial connections within the body or to the outside world. When this interface is changed dynamically in response to the neurophysiologic state, we call it *adaptive neuromodulation* (Lance et al., 2012; Wolpaw and Wolpaw, 2012; Birmingham et al., 2014). Adaptive neuromodulation has emerged as a powerful tool to influence short- and long-term nervous system activity and resulting behavior, opening entirely new ways to study brain function and to treat neurological disorders. It is currently in clinical practice or testing for: deep-brain stimulation (DBS) to treat Parkinson's disease (Arlotti et al., 2016; Little et al., 2016; Tinkhauser et al., 2017; Swann et al., 2018; Bouthour et al., 2019; Molina et al., 2021; Wu et al., 2021), essential tremor (Opri et al., 2020), or obsessive-compulsive disorder (OCD) (Alonso et al., 2015; Corva et al., 2021); implanted devices that detect the onset of a seizure and provide cortical or peripheral nerve stimulation to abort it (Handforth et al., 1998; Morrell, 2011; Rønborg et al., 2021; Wu et al., 2021); brain–computer interfaces (BCIs) that enable people to use brain signals (rather than muscles) for communication and control (Wolpaw and McFarland, 2004); direct cortical stimulation for the treatment of Tourette's syndrome (Molina et al., 2018; Xu et al., 2020); stimulation protocols to enhance functional recovery after stroke (Wang et al., 2010; Bundy et al., 2017; Foong et al., 2019); and systems that can improve walking after incomplete spinal cord injury (Thompson and Wolpaw, 2014; Capogrosso et al., 2016; Rowald et al., 2022). Adaptive stimulation could also improve the efficacy or reduce the side-effects of more conventional neuromodulation techniques, such as vagus nerve stimulation (George et al., 2000; Ben-Menachem, 2002).

Adaptive neuromodulation is made possible by the confluence of scientific, technological, and clinical/commercial driving factors. The first factor includes the increasing scientific appreciation that *the nervous system is highly plastic* (Raineteau and Schwab, 2001; Wolpaw, 2010), and can be artificially influenced to change in the setting of neurological disorders that would not improve naturally. The second driving factor is *the increasing availability of technological components* that support complex real-time adaptive interactions with the nervous system at affordable costs (e.g., miniaturized and biocompatible sensing and stimulation devices and processing hardware). The third driving factor is the rapidly growing appreciation of the wide-ranging *clinical and commercial opportunities* for adaptive neuromodulation, prompting substantial commercial investments in research, development, and commercialization of adaptive neuromodulation technologies. However, with three exceptions (i.e., cochlear prostheses that improve hearing, chronic deep-brain stimulation that improves movements, and responsive neurostimulation that suppresses epileptic seizures), clinical applications of neuromodulation have remained confined to relatively limited laboratory demonstrations. The biggest challenge for further clinical development of invasive neuromodulation protocols in humans is the substantial depth and breadth of technical, experimental, clinical, and regulatory expertise that must come together to translate scientific understanding into a device available for human use.

### 1.2. Requirements for further progress

Adaptive neuromodulation research is currently in an early stage, with relatively modest knowledge about how different areas in the brain interact to produce specific behaviors, how these interactions are affected by disease, and how to electrically modulate them to normalize pathologic behavior. Indeed, for most indications, we know very little about the ideal targets for sensing or stimulation, the optimal adaptive protocol, or the best parameters of stimulation.

Consider the case we likely understand best: the thalamocortical circuits for movement and the disorders associated with them that we treat with DBS. While we know the neuronal projections and general stimulation effects of different areas that reduce tremors associated with Parkinson's disease or essential tremor, the specific mechanism for each and the reasons for variation in efficacy are still unknown (Follett et al., 2010; Buhmann et al., 2017).

For other neurological or psychiatric disorders (such as chronic pain or depression), knowledge of the underlying neural system is much less developed than for movement disorders. Perhaps because there are no straightforward animal models for these disorders, neuromodulation studies tend to be based on relatively poorly grounded hypotheses, therefore, human studies for treatment are essentially forced to be expensive multi-year experiments based on trial and error. This unfortunate reality demands, and in practice critically requires, a readily available and easy-to-use general-purpose adaptive neuromodulation platform. With such a platform, scientists or clinicians could rapidly test different hypotheses about the relationship of neural measurement or stimulation protocols with behavior and disease state.

Successful engagement in human invasive adaptive neuromodulation research is exceedingly complex and difficult: it requires substantial *technical expertise* (to assemble and properly integrate hardware components, to write software for them, and to develop appropriate signal processing algorithms); *neuroscience and clinical expertise* (to design appropriate adaptive protocols, to surgically implant neuromodulation hardware, and to properly monitor the outcome of the protocol in a correctly defined patient population); and *regulatory expertise* (to select technologies and protocols that have a realistic chance to pass the FDA approval process, and to prepare an investigational device exemption (IDE) application using them). Hence, the most critical and pressing need in adaptive neuromodulation research and development is the generation and wide dissemination of a comprehensive set of easy-to-use hardware, software, experimental/surgical protocols, and regulatory documents/templates, i.e., an *ecosystem for adaptive neuromodulation research*, that reduces the expertise, complexity, and time of successfully engaging in basic or clinical neuromodulation research.

In this context, it is important to recognize that there are serious tradeoffs that affect medical devices that are intended to treat a specific disorder, but research-focused neuromodulation systems as well. A medical device will prioritize usability and will only have the most minimal set of technical features that are necessary to implement a specific treatment protocol. It will likely be fully implanted, have only few channels, and limited abilities to process data or to transmit raw data to an external device in real time. In marked contrast, research-focused neuromodulation systems will prioritize technical flexibility and capability over usability. Thus, their function may be distributed between an implanted unit and external components such as a laptop, with continual real-time exchanges between them. Because of this reliance on external components, such systems may only be useful in certain situations (such as the intraoperative scenario, with immobile patients, or with certain research protocols).

These tradeoffs notwithstanding, the availability of an adaptive neuromodulation ecosystem would make it possible to: (1) much more effectively and efficiently derive a better understanding of the physiological and pathological characteristics of a particular neural system supporting behavior; (2) use this improved understanding to formulate more specific hypotheses about how neuromodulation protocols may be used to alleviate pathological symptoms; (3) validate these protocols in clinical studies in humans; and (4) use the results of these studies to iteratively refine and guide subsequent neuroscientific inquiry and clinical validation. Without such an ecosystem, research that will enable the development of new neuromodulation protocols for different neurological disorders will continue to be greatly impeded, and progress will likely continue to remain limited. Fortunately, several existing hardware systems could serve as constituent components of this desired ecosystem. They are listed in the next section.

### 1.3. Existing hardware devices

At present, only nine hardware devices have been specifically designed to satisfy the complex needs of neuromodulation experimentation in the human brain (Kohler et al., 2017; Borton et al., 2020; Table 1). We left out Medtronic's PC+S/RC+S systems, because they are no longer available, but are including Cleveland's networked neuroprosthesis in this list. Moreover, we left out the existing implanted devices for neuromodulation in humans that are focused on neuromodulation of peripheral targets (e.g., vagus nerve), because their technical or implant characteristics make them unsuitable for complex adaptive stimulation of targets in the brain. None of the listed devices currently satisfy all requirements of invasive human adaptive neuromodulation research. The DyNeuMo (Picostim) (Zamora et al., 2021) system is the closest conceptually to our proposed ecosystem, but is very limited in channel number and designed for a narrower set of applications. The 32-channel sensing and stimulation CorTec BrainInterchange (BIC) device is much more technically advanced than the Medtronic/Neuropace devices, and it has been developed and tested specifically to support a wide range of human research. However, it is clear that its powerful hardware functions need to be paired with appropriate and capable software.

**Table 1:** Current hardware devices designed for invasive human experimentation.

	**Link-R 32 (Ripple)**	**Link-S 16 (Ripple)**	**WIMAGINE (France) Mestais et al. (2015)**	**W-HERBS (Japan) Matsushita et al. (2018)**	**NNPS (Cleveland) Peckham and Ackermann (2009)**	**RNS (Neuropace) Sun et al. (2008)**	**PERCEPT (Medtronic) Jimenez-Shahed (2021)**	**DyNeuMo (Picostim) Zamora et al. (2021)**	**BIC (CorTec) Kohler et al. (2017)**
Multi-channel signal acquisition	yes	no	yes	yes	yes	partial	partial	partial	yes
Multi-channel stimulus generation	no	partial	no	no	partial	partial	partial	partial	yes
Multi-modal data acquisition	partial	partial	partial	partial	no	no	no	partial	partial
Strong computational capabilities	no	no	no	no	yes	partial	partial	partial	partial
Interactive capabilities	partial	partial	partial	partial	no	no	no	partial	partial
Current path to regulatory approval	no	no	no	no	partial	yes	yes	partial	yes

Green: yes, yellow: partial, red: no.

## 2. Methods

### 2.1. The components of our platform

A general-purpose platform to optimally address the needs of invasive human neuromodulation research needs to be comprised of hardware and software that are both capable enough to facilitate research using a wide range of protocols. This platform also needs to provide the necessary configurations and documentation that minimize the time, complexity, and cost of implementing a particular research protocol. To forge our platform, we integrated the general-purpose and open-source software environment BCI2000 with CorTec's 32-channel sensing and stimulation device BrainInterchange.

### 2.2. Neuromodulation hardware: CorTec's BrainInterchange device

**Figure 1:**
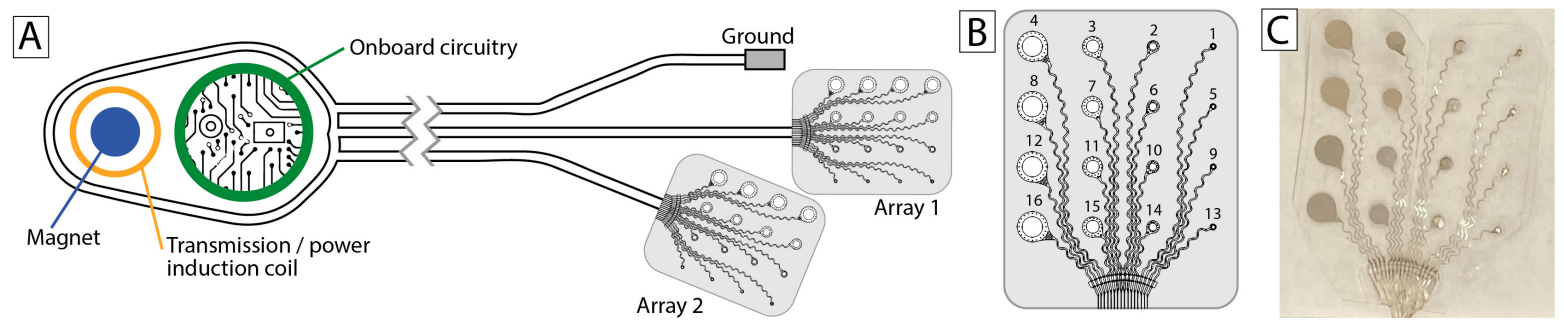
Implants. **(A)** CorTec's BrainInterchange (BIC) system. **(B)** The configuration of each of the two 16-channel multiscale AirRay cortical arrays (CorTec nr. 1015.5046.00) used for the initial implant. **(C)** Peri-operative photograph of one of the arrays.

CorTec's adaptive neuromodulation system [Brain Interchange (BIC), Figure 1] is specifically designed to address the need for complex and flexible human experimentation, and has been developed over more than a decade (Gierthmuehlen et al., 2014; Kohler et al., 2017). The BIC device consists of an internal electronics unit that records and stimulates, and an external unit that provides inductive power and communicates with an external computer. BIC supports 32 channels that are sampled at 1 kHz (and communicated at full bandwidth to the external computer) and are digitized at 16 bit (74 nV resolution). Sensing can be hardware-referenced to any individual channel or set of channels. Stimulation can be directed to any of the 32 electrodes and supports trains of up to 200 Hz (10 μs to 2.5 ms stimulus duration). Stimulation artifacts are not mitigated in hardware, but effects can vary widely depending on stimulation and electrode configurations. In accord with FDA requirements, the BIC has an array of safety features (e.g., thermal monitoring, limitation of stimulation current/voltage, charge balancing, galvanic barrier, integrity test of firmware), and other important features (e.g., three types of artifact suppression and data encryption).

The BIC system is complemented by CorTec's FDA-approved AirRay multi-electrode arrays (Gierthmuehlen et al., 2014) for recording from the surface of the brain (Figure 1B).

### 2.3. Neuromodulation software: The BCI2000 software platform

BCI2000 is a general-purpose software platform for closed-loop neuromodulation and similar experiments (Schalk et al., 2004; Schalk and Mellinger, 2010), and has been in active development for 22 years. Over this period, BCI2000 has supported experiments reported in more than 1,000 peer-reviewed publications (Brunner and Schalk, 2018), including many of the most influential studies in adaptive neurotechnology research (e.g., Leuthardt et al., 2004; Wolpaw and McFarland, 2004; Miller et al., 2010).

BCI2000 can be used to record signals from the brain, process them in meaningful ways, and use the outputs to determine the timing or nature of the feedback to the brain through sensory or electrical stimulation. These functions are highly adaptable, and perform well even in demanding situations (e.g., recording from 256 channels at high sampling rates).

In the context of neuromodulation experiments described here, BCI2000 provides many useful functions. For example, it can:

- Acquire all brain signals, including all relevant device measurements such as temperature, experimental events, such as stimulation timing, etc.
- Synchronize behavioral measurements acquired from many supported devices, such as eye trackers, data gloves, or wearable movement sensors.
- Re-reference brain signals in software.
- Calculate spectral amplitude/power/phase using different algorithms (e.g., bandpass-filtering and Hilbert transform, FFT, or AR spectral estimation).
- Classify the results of these measurements using linear classifiers.
- Adaptively track the output of these measurements over time.
- Provide auditory/visual/electrical stimulation based on specific timing protocols, or contingent on the results of brain signal or behavioral measurements.

These capabilities can be accessed without any external programs, or can be readily extended through robust and documented interfaces in C++, Python, and Matlab. The same filtering capabilities can be applied to online brain signal data as well as to offline data analyses, which facilitates algorithm optimizations, and there are extensive scripting capabilities that facilitate generation of complex and fully automated experimental protocols.

BCI2000 is highly optimized for performance, supporting rapid feedback with low latencies and low latency variations even in demanding experimental situations (Wilson et al., 2010). For example, in optimized configurations, jitter on audio output is less than 1 ms, and latency of electrical stimulation is less than 3 ms. BCI2000 comes with a fully documented timing certification system that determines system timing for any BCI2000 hardware/software configuration.

In work leading up to the present report, we incorporated full support for CorTec's BrainInterchange device into BCI2000.

### 2.4. FDA regulatory compliance pathway

BrainInterchange is a device that is designed to be chronically implanted in humans. As such, it is a Class III medical device, and its use is regulated by the FDA. Any research group that is interested in using the BrainInterchange device for their study needs to apply for an investigational device exemption (IDE) with the FDA.

CorTec is facilitating this IDE application process. They already completed all necessary tests that together ensure the safety of the device's use in humans. These tests include: technical validation, validation of the packaging process, transport validation and accelerated aging, validation of the cleaning and sterilization processes, mechanical testing, electrical testing, long-term stability testing, and biological–toxicological testing. CorTec used the results of these tests to establish a Master File with the FDA and will provide access to that Master File to the IDE applicant through a Rights of Reference letter.

### 2.5. Modeling the beagle skull and brain

**Figure 2:**
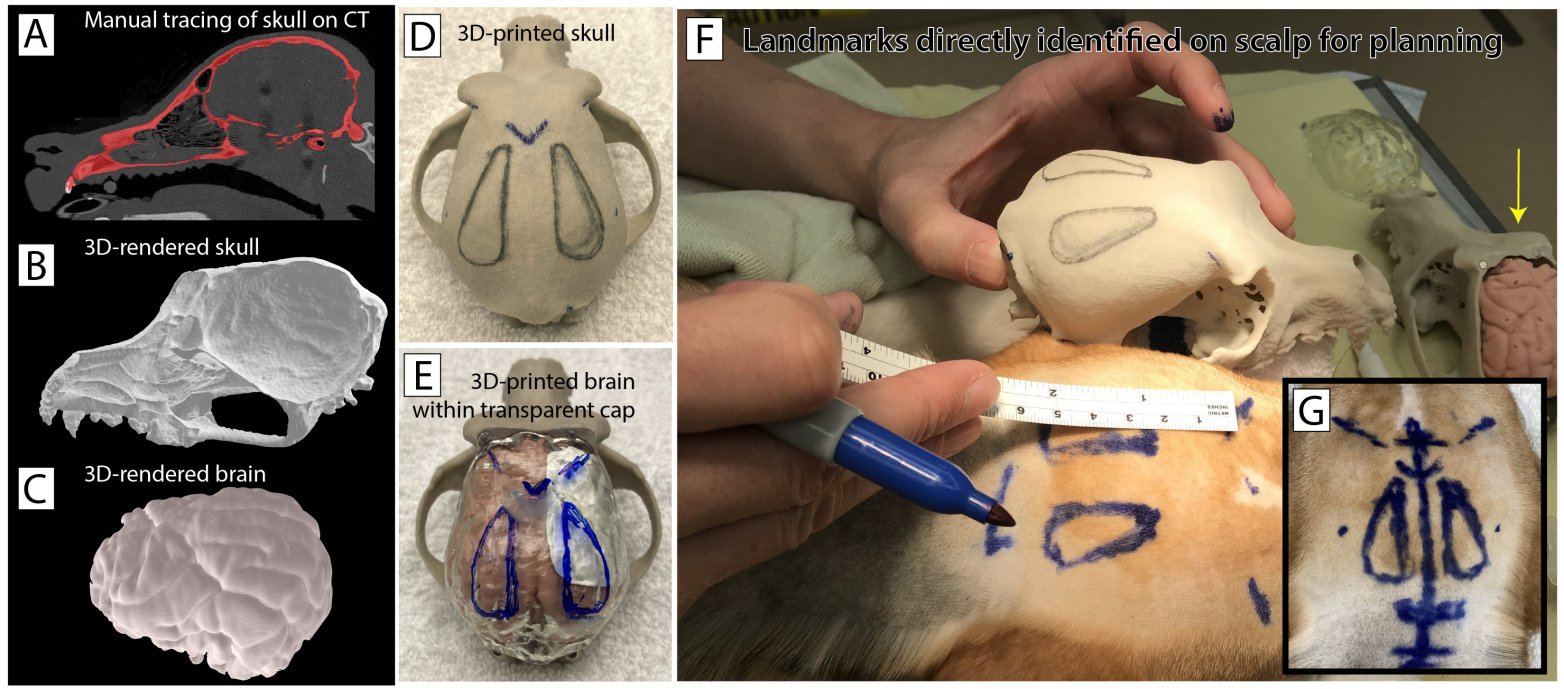
Modeling the individual canine anatomy for operative planning. **(A)** A pre-operative CT scan was obtained and the skull was traced out manually on individual slices. **(B)** The tracing from the CT scan was used to render a 3-dimensional skull as an .stl file. **(C)** A 3T T1 MRI sequence was used to trace out and render the beagle's brain in a similar fashion. **(D)** The skull was then printed in 3D ceramic at full scale. **(E)** A rubberized brain model was also printed along with a transparent cap. This allowed planning for the craniotomy relative to palpable skull landmarks, and the intended region of the grid (white paper on the brain model beneath the transparent cap). **(F,G)** These models were used in pre-operative planning to identify and mark skull landmarks and plan for surgical implantation directly on the animal's scalp. Note the rubberized brain model *in situ* in the top right (yellow arrow).

The pre-surgical workup included extensive high-resolution imaging (MRI, DTI, fMRI, and CT). Some of these images were the basis for different configurations of life-size high-resolution 3D-printed models to aid in surgical planning. To generate these models, we used a CT scan and MRI to manually trace out the skull and brain contours, and to render them in 3D (Figures 2A–C). We then printed precise full-scale replica models of the brain and skull at Mayo's *3D Anatomic Modeling Laboratories*^1^ (Figures 2D,E). Immediately before surgery, we identified precise landmarks on the beagle's scalp using these models as direct side-by-side reference to guide the craniotomy (Figures 2F,G).

### 2.6. Ethics statement

This research was conducted under Mayo Clinic IACUC protocol A00001713-16-R19. We maintain our canines in an IACUC-approved environment over their natural lifetime. In addition, according to State of Minnesota statute 135A.191, the canines can be made available for adoption if for any reason the research were to be discontinued. In the event of illness or decline, the animal will be euthanized according to an IACUC-approved protocol. The intent of this animal research is to test and develop a platform for novel human therapeutics.

## 3. Results

The following sections describe the first steps in developing and validating our ecosystem by integrating BrainInterchange hardware and BCI2000 software, and by implanting and testing this initial version in a beagle canine.

### 3.1. Operative implantation

The CorTec BrainInterchange device was implanted in a female beagle (10 kg body weight, fully grown, 2+ years old).

On the day of implantation, the animal was placed under general anesthesia by the veterinary staff, and positioned lying on her abdomen with forelimbs anterior, hindlimbs posterior, and head supported in a neutral position. The 3D models were used to determine skull landmarks, planned craniotomy, and planned midline incision in the scalp (Figures 2F,G). A second small incision was planned and marked immediately superior and posterior to the margin of the scapula of the right forelimb. After confirming all operative instruments and implanted hardware, the animal was prepped with sterile solution and draped with a sterile field.

**Figure 3:**
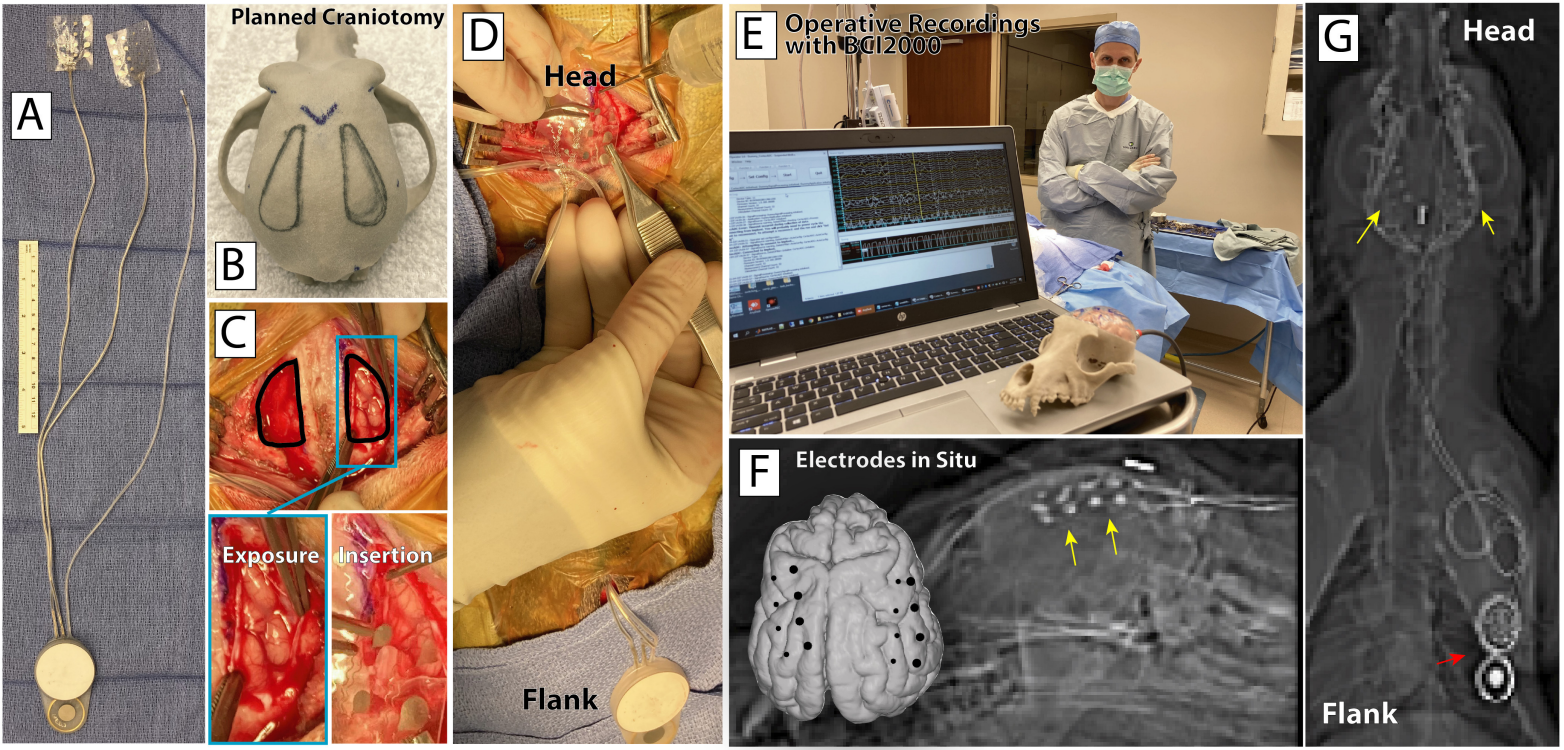
Surgical device implantation. **(A)** CorTec's BIC device and two 16-channel AirRay cortical electrodes. **(B)** Planned bilateral craniotomy on a 3D-printed model of the canine's skull. **(C)** Bilateral craniotomy, exposed cortex, and electrode grid insertion. **(D)** AirRay electrodes implantation in right subdural space, with tunneling of wires to BIC device at incision superior and posterior to the right scapula. **(E)** Successful implant and first online recordings from the implanted electrodes in the OR. **(F)** Co-registered pre-operative MRI and post-operative CT highlight the locations of the electrodes on the rendered brain (foreground) and on lateral radiograph (background). Yellow arrows highlight implanted electrodes. **(G)** Post-operative posterior-anterior radiograph showing the location of the electrodes, connecting wires, and BIC device.

Guided by our delineations of the craniotomies on a 3D model (Figures 3A,B), beginning at the head, an ~6 cm linear anterior-posterior incision was made in the scalp. The posterior portion of the temporalis muscle and fascia was elevated from the bone, and two small bone windows were made. A several centimeter incision was then made at the planned scapular site in the flank above the right forelimb. A tunneling rod was passed from the head incision to the flank incision, and then used to tunnel the arrays and wires of the device from the flank to the head. Small incisions were made in the dura bilaterally to expose the brain surface, and the electrode arrays were slid over the brain surface (Figures 3C,D). Due to the limited size of the exposure, it was necessary to cut two of the 4-contact rows on the left array and one 4-contact row on the right array to enable placement on the brain surface. The dura was sutured closed over the arrays, and a small plate with two screws was used on each side to hold the wires connected to each array fixed at the edge of the craniotomy, and provide strain relief. The temporalis fascia from each side was sewn together at the midline, protecting the craniotomy sites and holding the temporalis suspended at the top. The ground electrode was sutured in place at this confluence. Skin was closed with interrupted suture in each site. Post-operative antibiotics where administered to prevent infection. Verification of proper functioning of the implant electrodes and recording/stimulation hardware was achieved intraoperatively (Figure 3E). A post-operative CT scan was fused to the pre-operative MRI, allowing for direct localization of the electrode positions on the dog's brain surface (Figures 3F,G).

### 3.2. Experience with BCI2000 software integration

In work leading up to this study, we developed an initial version of a BCI2000 module that supports CorTec's BrainInterchange. This software module is fully documented on the BCI2000 wiki^2^. It currently supports recording of brain signals as well as all implant variables (such as temperature, humidity) from BrainInterchange, visualize raw data with different filtering options, provide real-time analyses (such as spectra), and, with proper configuration, provide electrical stimulation that is contingent on a specific brain state (e.g., a certain phase of a beta oscillation) or behavioral condition (e.g., a certain phase of walking).

### 3.3. Post-operative verification of recording capability

**Figure 4:**
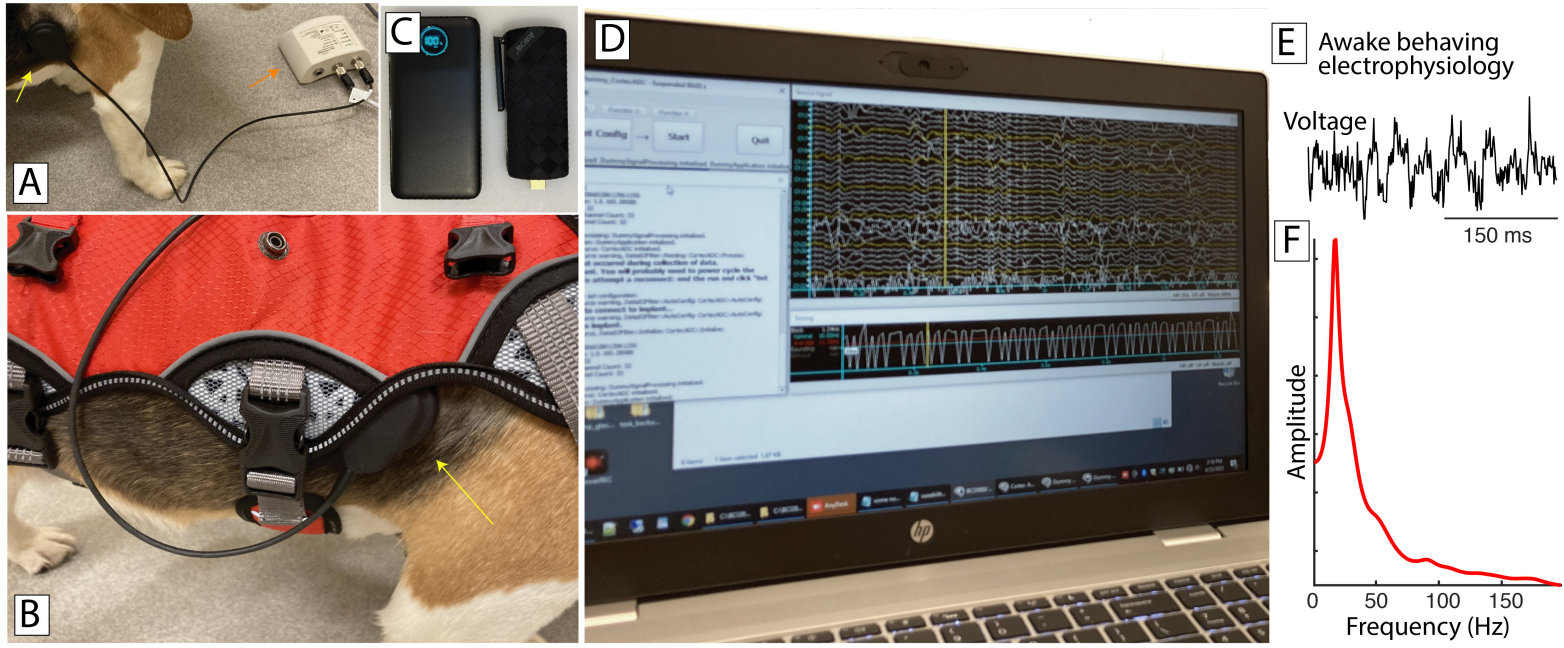
Awake recording environment. **(A)** The external component of the BIC device (red arrow) is connected to the implanted portion magnetically (yellow arrow). **(B)** A harness (red) can be put on, holding the external portion of the BIC along with a small computer and associated battery (e.g., in C), with the magnetically-coupled dongle interfacing with the implanted device (yellow arrow). **(C)** A small computer (right) and battery (left) run BCI2000 and interact with the BrainInterchange device. **(D)** A nearby laptop connects to and remotely controls BCI2000 on the small computer. **(E)** Postoperative ECoG activity in the awake behaving canine, recorded from an electrode over sensorimotor cortex. **(F)** Amplitude spectrum of this ECoG activity, revealing the expected oscillatory activity in the beta band.

Post-operatively, we used BCI2000 to record electrophysiological signals and implant parameters from the BIC device (Figure 4D). Electrophysiological signals were typical of electrocortical recordings (Figure 4E) and displayed the oscillatory activity commonly observed in motor cortical areas (Figure 4F; Miller et al., 2007).

The implant parameters appeared to be relatively stable over the period of recording (temperature 36.0–44.06° Celsius, implant voltage 6.0–6.5V, implant humidity 19.0–21.0%rh). Analyses demonstrated that about 1.9% of signal samples were lost in transmission, and that those samples were clustered around specific points in time.

## 4. Discussion

In this paper, we describe our initial work toward the development of a hardware/software ecosystem for adaptive neuromodulation research in humans that is based on CorTec's BrainInterchange implant hardware and BCI2000 software. Our experience demonstrates that we successfully implanted the BIC device and interfaced it with BCI2000, which exposes all technical capabilities of the BrainInterchange device to an experimenter. Thus, our successful demonstration brings us closer to the day when it will be possible to more easily directly interact with the human brain with sophisticated recording and stimulation protocols.

While our initial work is encouraging, it is still relatively early in development and validation, and lot of work remains to be done. For example, currently, the connection from BIC's external transmission unit to a computer is realized using a USB connection (Figure 4A). Thus, the initial configuration of our system would be useful only with completely immobile subjects such as patients with late-stage ALS or human/animal subjects in the operating room.

To address this issue, we began to develop a small system that can run BCI2000 interacting with BrainInterchange, and can do so for at least several hours without interruption (about 8 h in the configuration described here). This system consists of a portable battery and a mini PC stick that runs Windows 10 64-bit, has 4GB of RAM, 64GB SSD, an Intel Gemini Lake J4105 processor with enough computing power, and does not need a fan (Figure 4C). BIC's external transmission unit plugs into this computer and interacts with the BIC device. Thus, there are no range issues with a wireless link. The transmission unit, portable PC, and associated battery are light enough that they can be carried even by a medium-sized animal such as a canine using an appropriate harness (Figure 4B). While this harness proves useful, we still need to further improve this setup so that all cabling/connections remain in place while the animal is normally behaving (or even actively trying to manipulate them). In any case, the device can perform all of the recording/processing/visualization/stimulation functions BCI2000 can provide, connects to the local WiFi network, and can be remotely controlled using remote-control software.

We anticipate that, with completion of this development, with full technical and clinical validation, and with development of technical, clinical, and regulatory protocols, our work will create the first comprehensive ecosystem for adaptive neurotechnology research in humans that should make it easier for research groups to develop new neuromodulation protocols that address the devastating effects of different neurological disorders.

## Data availability statement

The original contributions presented in the study are included in the article/supplementary material, further inquiries can be directed to the corresponding author.

## Ethics statement

The animal study was reviewed and approved by Mayo Clinic IACUC protocol A00001713-16-R19. Written informed consent was obtained from the individual(s) for the publication of any potentially identifiable images or data included in this article.

## Author contributions

GS, SW, FM, GW, and KM: data recording and electrophysiology. SM and TK: neuroimaging. PB and AB: software development. DH: processing of neuroimaging. JM: 3D modeling. KM, BK, and NS: implant planning. IK, GW, and KM: animal preparation. GS, JR, and PB: hardware and software provisioning. PB, GW, and KM: funding acquisition. GS and KM: manuscript writing. All authors contributed to the article and approved the submitted version.

## Footnotes

^1^ https://www.mayoclinic.org/departments-centers/anatomic-modeling-laboratories/overview/ovc-20473121

^2^ https://www.bci2000.org/mediawiki/index.php/CortecADC
